# Silencing of insulin-like growth factor-1 receptor enhances the radiation sensitivity of human esophageal squamous cell carcinoma *in vitro* and *in vivo*

**DOI:** 10.1186/1477-7819-12-325

**Published:** 2014-11-03

**Authors:** Hui Zhao, Xiaomeng Gu

**Affiliations:** Department of Thoracic Surgery, Qilu Hospital of Shandong University, Wenhua Western Road 107, Jinan, 250012 Shandong Province China; Department of Gastroenterology, Qilu Hospital of Shandong University, Wenhua Western Road 107, Jinan, 250012 Shandong Province China

**Keywords:** Human esophageal squamous cell carcinoma, Radiation sensitivity, *IGF-1r*, RNAi

## Abstract

**Background:**

Esophageal squamous cell carcinoma (ESCC) is a prevalent fatal cancer worldwide, and the number of deaths due to this disease is increasing. Due to ESCC resistance to chemotherapy and radiation treatment, new therapies are urgently needed for the improvement of ESCC patient clinical outcomes.

**Methods:**

Eca-109 and TE-1 cells were transfected with 100 nM *IGF-1r* siRNA, and a combination of *IGF-1r* siRNA and radiation therapy was tested *in vitro* and *in vivo*. The effects of *IGF-1r* siRNA were determined through Western blotting and flow cytometry experiments.

**Results:**

After radiotherapy, the number of *IGF-1r* siRNA-transfected Eca-109 cells decreased by approximately 67.3%, and a 78.9% reduction was observed in the transfected TE-1 cells. In addition, the Eca-109 and TE-1 cells that were irradiated following *IGF-1r* knockdown contained 16.2% and 20.3% apoptotic cells, respectively.

**Conclusions:**

The results of the current study suggest that *IGF-1r* knockdown may enhance the radiation sensitivity of ESCC and increase the therapeutic effects of radiation both *in vitro* and *in vivo.* These results provide strong evidence that the targeted application of siRNA will enable the development of new therapeutic strategies for the clinical treatment of ESCC patients.

## Background

Esophageal squamous cell carcinoma (ESCC), one of the most common fatal cancers worldwide, is frequently diagnosed in China, and the number of deaths due to this disease is increasing [1–3]. Surgical resection is a standard treatment for ESCC patients, but its success depends on the advanced stage and development of the tumor. Further, chemotherapy and radiotherapy are also traditional therapeutic methods employed for the treatment of ESCC, often in combination. Unfortunately, although such multi-therapeutic strategies have been used for the treatment of ESCC patients, the overall five-year survival rate remains low at between 10 and 30% [4, 5]. In addition, the resistance of tumors to radiotherapy and chemotherapy has also been reported [6–8].

Thus, new therapeutic methods are urgently needed for the improvement of ESCC clinical treatment. It has been demonstrated that some pathogenic factors, such as cancer-related genes, are related to the progression and development of ESCC. Abnormal gene expression or modifications including certain genetic or epigenetic alterations, such as those found in tumor-suppressor genes or oncogenes, may cause tumor initiation [9, 10]. The data suggest that some related tumor genes can also increase the therapeutic efficacy of ESCC treatment by improving the chemotherapy or radiation sensitivity of tumors [11, 12]. However, the details and underlying mechanism of this effect are still unclear.

It was recently reported that insulin-like growth factor 1 (*IGF-1*), which can bind to *IGF-1* receptor (*IGF-1r*) to activate phosphoinositide-3-kinase and activate both the phosphoinositide-dependent protein kinase-1 (PDK1) and mammalian targets of rapamycin (mTOR) rictor kinases, is involved in tumor development, including progression and angiogenesis. *IGF-1* is thus a potential target in cancer therapy [13–16]. However, whether *IGF-1r* can modulate ESCC tumor sensitivity to chemotherapy or radiation therapy, has not been reported.

Small interfering RNA (siRNA), a recently developed technology, has been used to disrupt gene expression, especially of oncogenes or tumor-suppressors, which regulate target genes [17, 18]. However, to date, no evidence has been reported for the combination of radiation therapy and *IGF-1r* silencing in the treatment of ESCC. Therefore, *IGF-1r* siRNA combined with irradiation may be a potential therapeutic option for ESCC treatment. In the current study, it was hypothesized that radiation sensitivity will be enhanced after effective inhibition of *IGF-1r* through siRNA gene-silencing technology, which will result in a higher therapeutic efficacy in treating ESCC patients.

## Methods

### Cell lines

The human esophageal cancer cell lines Eca-109 and TE-1 were obtained from the American Type Culture Collection (Manassas, Virginia, United States). The cells were grown in Dulbecco’s modified Eagle’s Medium (DMEM) supplemented with 10% fetal calf serum (FCS) and 1% penicillin and streptomycin (Sigma-Aldrich, St. Louis, Missouri, United States). The cells were passaged every two to three days to maintain exponential growth prior to experimental usage and were maintained in 5% CO_2_ at 37°C.

### siRNA transfection

Eca-109 and TE-1 cells were transfected with 100 nM *IGF-1r* siRNA or a negative control vector (Qiagen, Lafayette, Colorado, United States) using Lipofectamine™ 2000 transfection reagent (Invitrogen, Carlsbad, California, United States) after the above cells were grown to between 75 and 85% confluency to obtain a higher transfection efficiency. After eight hours of transfection, the cell culture medium was replaced with DMEM. The *IGF-1r* gene targeting sequences were as follows: 5ʹ-ATTGAGGAGGTCACAGAGAAC-3ʹ and 5ʹ-TTCATATCCTGTTTTGGCCTG-3ʹ.

### Radiation treatment

After being subjected to *IGF-1r* siRNA transfection, which was performed as described above, both the Eca-109 and TE-1 cells received radiation treatment with γ-irradiation at a single dose of 4 Gy/min every three days in the presence or absence of *IGF-1r* siRNA.

### Western blotting

Western blotting was used to detect *IGF-1r* expression after *IGF-1r* siRNA transfection for 72 hours to evaluate the siRNA transfection efficiency in Eca-109 and TE-1 cells. At 24 hours after transfection, the medium was changed to serum-free medium. After a 72-hour transfection period, the cells were harvested, and cell lysates prepared in a buffer containing 0.1 M NaCl, 1 mM EDTA (ethylenediaminetetra-acetate, pH 8.0), 0.01 M Tris-HCl (pH 7.6), 1% (w/v) NP-40 (Nonidet P-40, octylphenoxy- polyethoxyethanol), 1% (w/v) Triton X-100, and 100 mg/ml PMSF(phenylmethanesulfonyl fluoride) (Sigma-Aldrich, St. Louis, Missouri, United States). Total protein was quantified by a Lowry protein concentration assay (Suolai, Beijing, China) after centrifugation at 12,000 × g for 60 minutes at 4°C. Equal amounts of protein were separated by SDS-PAGE and electrically transferred onto a PVDF (polyvinylidene fluoride) membrane. After blocking, the membrane was incubated overnight at 4°C with a primary antibody against *IGF-1r* (1:1,000; Santa Cruz Biotechnology, Santa Cruz, California, United States), followed by horseradish peroxidase-conjugated secondary antibody. The enhanced chemiluminescence system ECL-Plus (Suolai, Beijing, China) was used to detect the immunopositive bands, and the blot was stripped and re-probed using an antibody against β-actin (Sigma-Aldrich, St. Louis, Missouri, United States).

### Cell proliferation assay

In the next experiments, cell proliferation was evaluated by the method of 3-(4,5-dimethylthiazol-2-yl)-2,5-diphenyltetrazolium bromide. Briefly, Eca-109 and TE-1 cells were cultured in triplicate in 96-well plates at a density of 5 × 10^3^ cells/well. The cells were transfected with *IGF-1r* siRNA and received the following irradiation treatment as described above. Cells in each treatment group were harvested by trypsinization, and the cell growth was analyzed by a Universal Microplate Spectrophotometer (BioTek Instruments, Winooski, Vermont, United States).

### Analysis of apoptotic cells by flow cytometry

Eca-109 and TE-1 cells were transfected with *IGF-1r* siRNA and received the following irradiation treatment as described above. Cell apoptosis was evaluated through staining with propidium iodide and Annexin V-FITC using flow cytometry (Becton Dickinson, San Jose, California, United States).

### *In vivo*tumor xenograft studies

An animal model with subcutaneous tumor xenografts was established by injecting Eca-109 and TE-1 cells into the left dorsal flank (1 × 10^5^ cells per animal) of female nude mice (between six and eight-weeks-old) (Shandong University, Jinan, China). After 14 days, the above animals received the irradiation treatment in the presence or absence of *IGF-1r* siRNA transfection. Student’s t-test was used to determine the statistical significance of the therapeutic effects. All procedures were approved by the Animal Ethics Committee of Shandong University (QL-2012JMK-231).

## Results

### Evaluation of *IGF-1r*siRNA transfection efficiency

**Figure 1:**
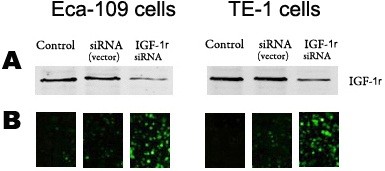
**Transfection efficiency of** ***IGF-1r*** **siRNA** ***in vitro*** **. (A)** *IGF-1r* protein expression was analyzed by Western blotting after transfection with *IGF-1r* siRNA for 72 hours in TE-1 and Eca-109 cells. **(B)** The transfection efficiency of *IGF-1r* siRNA was observed by fluorescence microscopy after 72 hours in TE-1 and Eca-109 cells. β-actin was used as a loading control.

Western blotting was used to test the expression level of *IGF-1r* to evaluate the transfection efficiency of the *IGF-1r* siRNA. It was confirmed that the expression of *IGF-1r* decreased by approximately 33% and 46% in the Eca-109 and TE-1 cells, respectively, after *IGF-1r* siRNA transfection for 72 hours. In addition, a difference in *IGF-1r* expression between Eca-109 and TE-1 cells was also observed, with *IGF-1r* expression being much higher in TE-1 cells than in Eca-109 cells (Figure 1A).

Furthermore, the *IGF-1r* siRNA transfection efficiency was also evaluated with green fluorescence analysis using a fluorescence microscope (OLYMPUS, Tokyo, Japan). The results showed that the level of green fluorescence was greatly increased, which confirmed that effective transfection was obtained after 72 hours of siRNA transfection both in Eca-109 and TE-1 cells (Figure 1B).

### Effective inhibition of cell proliferation after combination treatment

**Figure 2:**
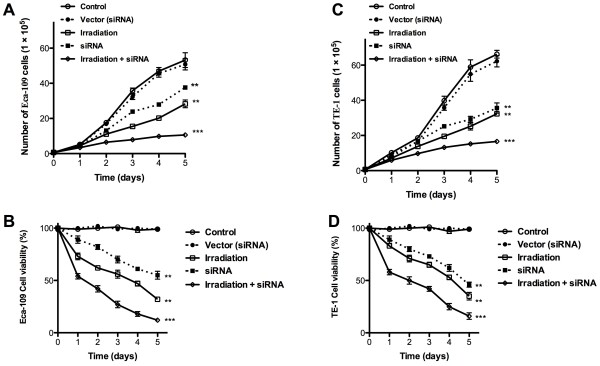
**Effect of combination therapy on the growth of esophageal squamous cell carcinoma cell lines** ***in vitro*** **. (A** **, B)** Cell proliferation of Eca-109 cells was influenced after γ-irradiation at 4 Gy/min (IR) in the presence or absence of *IGF-1r* siRNA. **(C** **, D)** Cell proliferation of TE-1 cells was influenced after γ-irradiation at 4 Gy/min (IR) in the presence or absence of *IGF-1r* siRNA. Bars indicate the standard deviation of the mean. Each experiment was performed in triplicate. (The criterion for statistical significance was *, ** and *** as p < 0.05, 0.01 and 0.001, respectively).

To evaluate the effect of radiotherapy on cell proliferation in the presence or absence of *IGF-1r* siRNA transfection *in vitro*, the cell proliferation of Eca-109 and TE-1 cells was determined in the following experiment. The control group was treated with *IGF-1r* siRNA or with radiation alone. The controls were compared to cells receiving a combination of γ-irradiation therapy of 4 Gy/min and *IGF-1r* siRNA transfection for 72 hours. Both radiation and *IGF-1r* siRNA can effectively inhibit cell proliferation alone, with radiation reducing the cell counts by 42.1% and 45.1% and *IGF-1r* siRNA reducing them by 36.5% and 43.1% in Eca-109 and TE-1 cells, respectively, compared to the untreated group (*P* <0.05, n *=*6). For the group receiving the combination therapy, the inhibition of cell proliferation was also more effective than that in the control group for both Eca-109 and TE-1 cells (*P* <0.001, n =6). After radiotherapy, the number of Eca-109 cells decreased by approximately 67.3% in the presence of *IGF-1r* siRNA transfection, and there was a 78.9% reduction in the TE-1 cells after radiation (Figure 2). In the TE-1 cells, the inhibition of cell proliferation after *IGF-1r* siRNA transfection was much higher than in the Eca-109 cells (*P* <0.01, n =6); this difference may result from the different expression levels of *IGF-1r* in these two cell lines. These results also show that the inhibition of cell proliferation after *IGF-1r* siRNA or radiation treatment alone was lower than in the combination treated group.

### Effects of combination treatment on apoptosis

**Figure 3:**
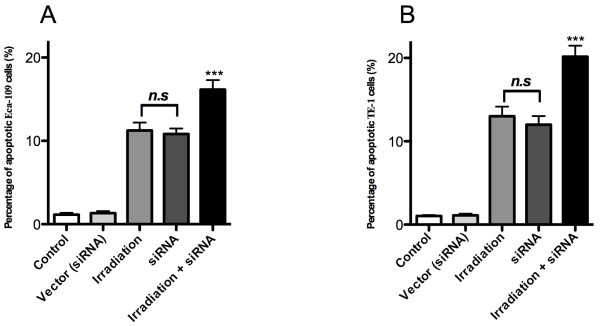
**Effect of combination therapy on apoptosis in TE-1 and Eca-109 cells. (A)** Eca-109 and **(B)** TE-1 cells were γ-irradiated at 4 Gy/min in the presence or absence of *IGF-1r* siRNA and then evaluated by flow cytometry. Bars indicate the standard deviation of the mean. Each experiment was performed in triplicate.

To test the effect of *IGF-1r* knockdown on Eca-109 and TE-1 cell apoptosis, flow cytometry was used to investigate the percentage of apoptotic cells. The percentage of apoptosis was 11.2 ± 0.8% after irradiation alone in Eca-109 cells. In the combination group, 16.2 ± 1.1% apoptotic Eca-109 cells were observed after irradiation coupled with *IGF-1r* siRNA treatment. In the TE-1 cells, 13.1 ± 1.1% apoptotic cells were observed after irradiation alone, and 20.3 ± 2.1% apoptotic cells were observed in the presence of *IGF-1r* siRNA treatment following irradiation, respectively (Figure 3). This result also suggests that some differences in apoptosis were observed between the Eca-109 and TE-1 cells (*P* <0.01, n =6), and these data were also consistent with the results that showed a lower expression of *IGF-1r* in Eca-109 cells than in TE-1 cells. Furthermore, it was clear that the percentage of apoptotic cells increased significantly after combination treatment compared to the cells that only received radiation (*P* <0.01, n =6).

### Effects of combination therapy on subcutaneous tumor growth

**Figure 4:**
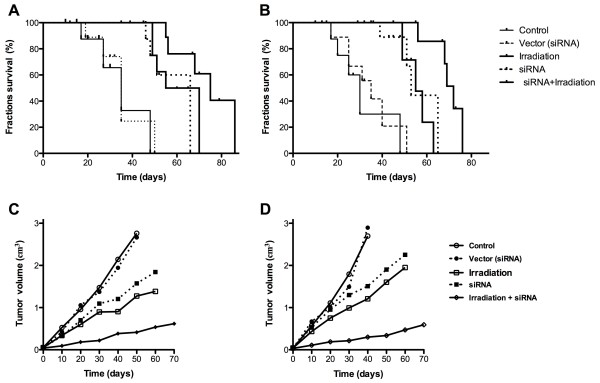
**Therapeutic effect of irradiation combined with** ***IGF-1r*** **siRNA** ***in vivo*** **.** The survival fraction after treatment is shown for **(A)** Eca-109 and **(B)** TE-1 cells, respectively. Irradiation alone enhanced tumor growth inhibition and led to shorter survival times than the combination treatment. The tumor volume was calculated at the indicated times in the **(C)** Eca-109 and **(D)** TE-1 cell groups, respectively. The statistical significance of the tumor volume changes was calculated using Student’s t-test.

The therapeutic efficacy of combination treatment was also investigated through *in vivo* tumor xenograft studies after injecting Eca-109 and TE-1 cells. The tumor volumes and survival rates were evaluated 14 days after cell injection in the different treated groups. The tumor volumes were 3.4 ± 0.2 cm^3^ in the Eca-109 group and 3.8 ± 0.1 cm^3^ in the TE-1 group after treatment with phosphate buffered saline (negative control group). After radiotherapy alone, the tumor volume decreased significantly both in the Eca-109 (2.1 ± 0.1 cm^3^) and TE-1 cells (2.4 ± 0.1 cm^3^) (*P* <0.05, n =8). In the group that received *IGF-1r* siRNA treatment alone, the tumor volume was also reduced (2.8 ± 0.1 cm^3^ for Eca-109 cells; 3.1 ± 0.1 cm^3^ for TE-1 cells) (*P* <0.05, n =8). Notably, it was clear that the tumors were much smaller after irradiation in the presence of *IGF-1r* siRNA transfection than in any single treatment group (1.1 ± 0.2 cm^3^ in the Eca-109 group; 0.8 ± 0.1 cm^3^ in the TE-1 group) (*P* <0.001, n =8) (Figure 4A). In addition, the survival rates of the tumor-bearing mice were also calculated after different therapeutic strategies. The results showed that the combination therapy significantly enhanced the survival rate compared to the groups with a single treatment (Figure 4B). These *in vivo* results confirmed that *IGF-1r* silencing can increase the radiation sensitivity of ESCC tumors in this established tumor model.

## Discussion

It has been reported that radiation resistance can reduce the therapeutic efficiency of irradiation in clinical ESCC tumor treatments, and thus, a new method that can increase the radiation sensitivity of tumor therapies is urgently needed to enhance the survival rate of ESCC patients. Overexpression of *IGF-1r* in tumors has been demonstrated in some reports [19, 20], so to test the hypothesis that *IGF-1r* silencing may increase the therapeutic efficacy of radiotherapy in the treatment of ESCC, a combination of *IGF-1r* siRNA and radiation therapy was tested *in vitro* and *in vivo*.

The following novel findings were demonstrated in our current study: (1) higher *IGF-1r* expression was observed in human TE-1 cells than in Eca-109 cells; (2) effective inhibition of cell proliferation and apoptosis in the presence of *IGF-1r* siRNA following radiotherapy was demonstrated *in vitro*; and (3) enhancement of tumor growth radiation sensitivity after *IGF-1r* siRNA treatment was observed *in vivo.* Thus, we demonstrated that greater therapeutic efficacy may be obtained by enhancing the sensitivity of tumors to radiotherapy in the presence of *IGF-1r* siRNA knockdown.

It was shown that higher *IGF-1r* expression was observed in human TE-1 cells than in Eca-109 cells, and the data suggested that *IGF-1r* deficiency can be obtained effectively in the above cell lines using the siRNA silencing method and that this method can be used to evaluate the effects of *IGF-1r* modulation on radiotherapy treatment. Results from both Western blotting and fluorescence microscopy demonstrated that effective *IGF-1r* siRNA transfection had been achieved in TE-1 and Eca-109 cells. However, different levels of *IGF-1r* expression were also observed in these cell lines, and the divergence of *IGF-1r* expression in these two cell types may explain the different clinical pathology characteristics of ESCC.

The therapeutic efficacy of irradiation on cell proliferation was dramatically enhanced in the presence of *IGF-1r* siRNA application *in vitro,* and this effect on the inhibition of cell proliferation was higher than the effect after radiation therapy alone. Cell apoptosis was also investigated to evaluate the effect of *IGF-1r* siRNA on cell sensitivity to irradiation. The effect of radiation therapy on cell apoptosis was also modulated after transfection with *IGF-1r* siRNA in TE-1 and Eca-109 cells, and a significant enhancement in the percentage of apoptotic cells was observed after combination therapy. An animal tumor model was established by subcutaneously injecting TE-1 or Eca-109 cells to test the anti-tumor effects of radiation therapy *in vivo* in the presence or absence of *IGF-1r* siRNA. Compared to the combination therapy group, larger tumor sizes and lower survival rates were obtained after radiation therapy in the absence of *IGF-1r* siRNA treatment. These results demonstrated that *IGF-1r* deficiency can enhance radiation sensitivity, but the underlying mechanism requires further investigation.

The *in vivo* and *in vitro* data clearly showed that the combination therapy had greater anti-tumor therapeutic efficacy in both the TE-1 and Eca-109 cells due to the enhancement of tumor radiation sensitivity in the presence of *IGF-1r* siRNA. Differences between the expression of *IGF-1r* in the TE-1 and Eca-109 cells were also observed, and these characteristics may affect the different responses to radiation treatment in ESCC patients with different pathological cell types.

## Conclusions

The current study demonstrated that *IGF-1r* siRNA, which can effectively reduce *IGF-1r* expression in both TE-1 and Eca-109 cells, may enhance the radiation sensitivity of ESCC tumors and increase the therapeutic effects of radiation both *in vitro* and *in vivo.* These results provide strong evidence that targeted use of siRNA may enable the development of new therapeutic strategies for the clinical treatment of ESCC patients.

## Abbreviations

DMEM: Dulbecco’s modified Eagle’s Medium
EDTA: ethylenediaminetetra-acetate
ESCC: Esophageal squamous cell carcinoma
FCS: fetal calf serum
IGF-1: insulin-like growth factor 1
PDK1: phosphoinositide-dependent protein kinase-1
mTOR: mammalian targets of rapamycin
NP-40: Nonidet P-40, octylphenoxy- polyethoxyethanol
PMSF: phenylmethanesulfonyl fluoride
PVDF: polyvinylidene fluoride
siRNA: Small interfering RNA.

## References

[1] Shang L, Wang M (2013). Molecular alterations and clinical relevance in esophageal squamous cell carcinoma. Front Med.

[2] Lin Y, Totsuka Y, He Y, Kikuchi S, Qiao Y, Ueda J, Wei W, Inoue M, Tanaka H (2013). Epidemiology of esophageal cancer in Japan and China. J Epidemiol.

[3] Thallinger CM, Kiesewetter B, Raderer M, Hejna M (2012). Pre- and postoperative treatment modalities for esophageal squamous cell carcinoma. Anticancer Res.

[4] Stoner GD, Wang LS, Chen T (2007). Chemoprevention of esophageal squamous cell carcinoma. Toxicol Appl Pharmacol.

[5] Nakajima M, Kato H (2013). Treatment options for esophageal squamous cell carcinoma. Expert Opin Pharmacother.

[6] Xie L, Song X, Yu J, Wei L, Song B, Wang X, Lv L (2009). Fractionated irradiation induced radio-resistant esophageal cancer EC109 cells seem to be more sensitive to chemotherapeutic drugs. J Exp Clin Cancer Res.

[7] Sugimura K, Miyata H, Tanaka K, Hamano R, Takahashi T, Kurokawa Y, Yamasaki M, Nakajima K, Takiguchi S, Mori M, Doki Y (2012). Let-7 expression is a significant determinant of response to chemotherapy through the regulation of IL-6/STAT3 pathway in esophageal squamous cell carcinoma. Clin Cancer Res.

[8] Wang XC, Tian LL, Tian J, Li D, Wang Y, Wu H, Zheng H, Meng AM (2012). Overexpression of Cks1 increases the radiotherapy resistance of esophageal squamous cell carcinoma. J Radiat Res.

[9] Chen J, Kwong DL, Cao T, Hu Q, Zhang L, Ming X, Chen J, Fu L, Guan X (2013). Esophageal squamous cell carcinoma (ESCC): advance in genomics and molecular genetics. Dis Esophagus.

[10] Bellini MF, Silva AE, Varella-Garcia M (2010). Genomic imbalances in esophageal squamous cell carcinoma identified by molecular cytogenetic techniques. Genet Mol Biol.

[11] He LR, Liu MZ, Li BK, Rao HL, Deng HX, Guan XY, Zeng YX, Xie D (2009). Overexpression of AIB1 predicts resistance to chemoradiotherapy and poor prognosis in patients with primary esophageal squamous cell carcinoma. Cancer Sci.

[12] Li SH, Huang EY, Lu HI, Huang WT, Yen CC, Huang WC, Chen CH (2012). Phosphorylated mammalian target of rapamycin expression is associated with the response to chemoradiotherapy in patients with esophageal squamous cell carcinoma. J Thorac Cardiovasc Surg.

[13] Arcaro A (2013). Targeting the insulin-like growth factor-1 receptor in human cancer. Front Pharmacol.

[14] Doyle SL, Donohoe CL, Finn SP, Howard JM, Lithander FE, Reynolds JV, Pidgeon GP, Lysaght J (2012). IGF-1 and its receptor in esophageal cancer: association with adenocarcinoma and visceral obesity. Am J Gastroenterol.

[15] Imsumran A, Adachi Y, Yamamoto H, Li R, Wang Y, Min Y, Piao W, Nosho K, Arimura Y, Shinomura Y, Hosokawa M, Lee CT, Carbone DP, Imai K (2007). Insulin - like growth factor-I receptor as a marker for prognosis and a therapeutic target in human esophageal squamous cell carcinoma. Carcinogenesis.

[16] Adachi Y, Ohashi H, Imsumran A, Yamamoto H, Matsunaga Y, Taniguchi H, Nosho K, Suzuki H, Sasaki Y, Arimura Y, Carbone DP, Imai K, Shinomura Y (2014). The effect of IGF-I receptor blockade for human esophageal squamous cell carcinoma and adenocarcinoma. Tumour Biol.

[17] Luo M, Shen D, Zhou X, Chen X, Wang W (2013). MicroRNA-497 is a potential prognostic marker in human cervical cancer and functions as a tumor suppressor by targeting the insulin-like growth factor 1 receptor. Surgery.

[18] Sukka-Ganesh B, Mohammed KA, Kaye F, Goldberg EP, Nasreen N (2013). Ephrin- A1 inhibits NSCLC tumor growth via induction of Cdx-2 a tumor suppressor gene. BMC Cancer.

[19] Fernandez MC, Martin A, Venara M, Calcagno Mde L, Sansó G, Quintana S, Chemes HE, Barontini M, Pennisi PA (2013). Overexpression of the insulin-like growth factor 1 receptor (IGF-1R) is associated with malignancy in familial pheochromocytomas and paragangliomas. Clin Endocrinol (Oxf).

[20] Mountzios G, Kostopoulos I, Kotoula V, Sfakianaki I, Fountzilas E, Markou K, Karasmanis I, Leva S, Angouridakis N, Vlachtsis K, Nikolaou A, Konstantinidis I, Fountzilas G (2013). Insulin-like growth factor 1 receptor (IGF1R) expression and survival in operable squamous-cell laryngeal cancer. PLoS One.

